# Longitudinal profiling of gut microbiome among tuberculosis patients under anti-tuberculosis treatment in China: protocol of a prospective cohort study

**DOI:** 10.1186/s12890-019-0981-9

**Published:** 2019-11-11

**Authors:** Wenpei Shi, Yi Hu, Xubin Zheng, Zhu Ning, Meiying Wu, Fan Xia, Stefanie Prast-Nielsen, Yue O. O. Hu, Biao Xu

**Affiliations:** 1 Department of Epidemiology, School of Public Health and Key Laboratory of Public Health Safety (Ministry of Education), Fudan University, Shanghai, China; 2 Zigong Center for Disease Control and Prevention, Zigong City, Sichuan China; 3 Department of Clinical Laboratory, The Fifth People’s Hospital of Suzhou, Infectious Disease Hospital Affiliated to Soochow University, Suzhou City, Jiangsu China; 4 Department of Tuberculosis, The Eighty-Fifth Hospital of Chinese PLA, Shanghai, China; 5 Center for Translational Microbiome Research (CTMR), Department of Microbiology, Tumor and Cell Biology, Karolinska Institutet, Stockholm, Sweden; 6 Department of Public Health Sciences, Karolinska Institutet, Stockholm, Sweden

**Keywords:** Gut microbiota, Pulmonary tuberculosis, Adverse effect, Treatment outcome, Chinese cohort, 16S rRNA gene sequencing

## Abstract

**Background:**

Anti-tuberculosis therapy requires at least six-month treatment with continuous administration of combined antibiotics, including isoniazid, rifampicin, pyrazinamide, and ethambutol. The long-term exposure to antibiotics could cause consequent changes in gut microbiota, which may alter the gastrointestinal function and drug absorption in patients, thereby affect the outcome of treatment. The study aims to characterize the longitudinal changes of gut microbiota among tuberculosis (TB) patients under standardized first-line treatment and provide an understanding of the association between alterations in gut microbiota composition and unfavorable clinical outcomes.

**Methods:**

The study is a multicenter, observational prospective cohort study. Three study sites are purposively selected in the western (Sichuan Province) and eastern (Jiangsu Province and Shanghai) parts of China. Three-hundred patients with bacteriologically confirmed pulmonary TB are enrolled. All eligible patients should be investigated using structured questionnaires before treatment initiation; and be followed up during the treatment at Day-14, Month-2, Month-5, the end of treatment and the sixth month after ending therapy. Stool samples are to be collected at each visit, consisting of six stool samples from each patient. Additionally, 60 healthy volunteers from Sichuan province and Shanghai city will be recruited as healthy controls to form the baseline of patient gut microbiota in the Chinese population. The dynamic changes of gut microbiota in terms of alpha diversity, beta diversity, taxonomic composition are to be illustrated individually from the time at diagnosis until the sixth month after therapy is completed. Furthermore, the diversity and component of gut microbiota will be compared between the groups with and without unfavorable treatment outcome in terms of adverse effect and treatment failure.

**Discussion:**

Studies on the clinical manifestations, adverse reactions, and gut microbiota alterations will provide scientifically-sound evidence on the impact of gut microbiota alterations on TB treatment outcomes. The study is not only useful for guiding personalized TB treatment but also sheds light on the effects of continuous antibiotics administration on gut microbiota.

**Trial registration:**

Chinese Clinical Trial Registry, trial ID: ChiCTR1900023369, May 24, 2019. Retrospectively registered.

## Background

Tuberculosis (TB), caused by *Mycobacterium tuberculosis* (*M. tb*), has plagued humans for thousands of years and remains a major public health problem. Estimated by the World Health Organization (WHO), there were 10 million TB cases and 1.6 million TB deaths worldwide in 2017 [1]. Among them, 890,000 new TB cases (8.9%) were infected in China, with a mortality rate of 26/100,000 [1]. TB is a curable disease, and the modern TB control program recommends the strategy of directly observed treatment – short-course therapy, which is a six-month standardized treatment using first-line anti-TB drugs (FLDs) including Isoniazid (INH), Rifampicin (RFP), Pyrazinamide (PZA), and Ethambutol (EMB). This potent standardized drug regimen has contributed to an up to 90% cure rate in TB patients during the last three decades. However, the high rate of recurrence and retreatment among TB patients, the ubiquity of severe gastrointestinal adverse reactions [1, 2], and the increasing epidemic of multidrug-resistant tuberculosis (MDR-TB) all suggest that the effectiveness of anti-TB treatment in China has yet to be addressed.

Therefore, introducing new knowledge and clinical strategies to improve the therapeutic effect and clinical prognostics in Chinese TB patients is of great significance. The recent research advances in gut microbiome have shown that these bacteria are not only actively involved in the absorption of food and drugs but also produce bioactive compounds influencing the immune and hormone systems, which is essential to maintain vital functions of the healthy host [3]. Generally, the human gut microbiota shows a large degree of interpersonal diversity [4], and its composition is also affected by many factors such as geography, diet, season, lifestyle, disease state, and in particular, the use of antibiotics [3, 5].

Mounting evidence has shown that antibiotics administration could result in the dysbiosis of gut microbiota which was associated with many clinical concerns including accumulation of resistance genes [6], increased susceptibility to pathogens [7, 8], and compromised immune homeostasis and tolerance [9]. Different antimicrobial agents can influence the gut microbiota in different ways. The widespread use of the FLDs using a mixture of broad-spectrum (RFP) and mycobacterial specific antibiotics (INH, PZA, EMB) for at least 6 months is a long-term, combined, and large dose antibiotic therapy. RFP has a broad-spectrum activity against a wide range of Gram-positive and Gram-negative bacteria [6]. Even for narrow-spectrum antibiotics, drugs might affect other microbes in the body, either directly by their antimicrobial activity or by host immune system modulation [10]. Referred to previous population-based studies, 20 to 25% of TB patients suffered from gastrointestinal adverse reactions and liver dysfunction during anti-TB treatment [11], which directly damaged the intestinal mucosa and led to the reduced drug absorption and liver metabolism [12].

Recent studies carried out in TB patients indicated that standardized first-line anti-TB treatment caused acute changes in the intestinal microbiota, although information on diversity changes and the altered taxa are limited. Very few studies had taken a prospective approach and most of these studies had a cross-sectional design with small sample sizes and potential heterogeneity in study participants [13–15]. Additionally, the aforementioned evidence only clarified the acute microbiome changes within the first 2 months of anti-TB treatment. As a therapy with at least 6 months of antibiotics administration, it is of great importance to systematically investigate the long-term effect of anti-TB treatment on patients’ gut microbiota and the potential influence of gut microbiota alterations on treatment outcomes and drug-induced adverse effects. Therefore, a well-controlled prospective study is needed to provide a better understanding of these questions.

## Methods/design

### Objectives

#### Primary objective

The primary objective of this study is to explore the effects of continuous administration of FLDs on the gut microbiota by analyzing its alterations on diversity and taxonomic composition in pulmonary TB patients in China.

#### Secondary objectives

1. To describe the baseline features of gut microbiota before anti-TB treatment among TB patients with different age, gender, health status, disease comorbidity in different regions of China.
2. To compare the characteristics of gut microbiota in terms of diversity and taxonomic composition between TB patients at baseline and healthy controls.
3. To describe the incidence of gut microbiota dysbiosis during anti-TB treatment and its association with adverse effects and unfavorable treatment outcomes in pulmonary TB patients through a case-control study nested in the conducted cohort.
4. To identify the potential microbial markers of unfavorable treatment outcomes.
5. To observe the long-term effect of TB treatment on intestinal microbiota after therapy is completed.

### Study design

This study is an ongoing multicenter, population-based prospective cohort study conducted in three TB designated hospitals in Eastern and Western China. The study aims to include 300–400 bacteriologically confirmed pulmonary TB patients. Baseline information, including demographics, socioeconomic status, laboratory tests, and disease profiles, as well as sputum and stool samples will be collected from the enrolled patients immediately after TB diagnosis. The patient cohort will be built and followed up during the first-line anti-TB treatment at Day-14, Month-2, Month-5, last day of treatment, and the sixth month after treatment. Information regarding adverse effects, disease progress, and laboratory results will be collected at each visit. Stool samples will be sent to the laboratory for 16S ribosomal ribonucleic acid (rRNA) gene sequencing to identify the alterations in gut microbiota. A comparison of gut microbiota will be performed between newly diagnosed TB patients and healthy controls to reflect the difference in baseline features. After treatment completion, a nested case-control study will be performed between patients with treatment success or failure and patients with or without adverse effects to screen the potential biomarkers from the gut microbiota indicative of therapeutic effect.

### Settings

Considering the differences in dietary habits, local economy and TB epidemic, patient recruitment for this study will take place at three geographic locations, i.e. Sichuan province, Jiangsu province, and Shanghai.

Shanghai (30°N 120°E) is an international metropolitan city in China with an estimated 24 million residents [16], first-ranked per capital disposable income and lowest TB report rate (14.75/100,000) in 2017 (Table 1) [17]. As one of the richest provinces in China, Jiangsu is located on the eastern coast of China next to Shanghai with the middle level of TB epidemic (33.52/100,000 [18]). On the contrary, Sichuan is an inland province located in Western China. Compared to the other two study sites, the income level, sanitation condition and health resources in Sichuan are less developed [16]. The notification rate of TB in Sichuan was 69.41 per 100,000 in 2017 [19], higher than the average level in China [20]. Regarding the dietary habits, Sichuan is famous for spicy food and high salt intake while people in Shanghai and Jiangsu prefer the light and sweet taste.

**Table 1:** Economy, health resources and tuberculosis epidemic in study sites in 2017

Study sites	Per capita GDP^a^($)	Per capita disposable income ($)	Medical technical personnel in health care institutions (per 1000 persons)	Notified TB^b^ incidence rate (per 100,000 persons)
Sichuan	6671	2980.12	6.39	69.41
Jiangsu	16,010	5071.75	6.82	33.52
Shanghai	18,931	8541.93	7.73	14.75

^a^*GDP* Gross domestic product, ^b^*TB* Tuberculosis

### Participants and recruitment

#### Patient cohort

Patients diagnosed with active pulmonary TB are eligible to participate in this study before their treatment initiation between July 2018 to December 2020. Pulmonary TB should be confirmed by acid-fast bacilli (AFB) smear microscopy, sputum culture, and chest radiograph. Conventional drug-susceptibility testing (DST) and advanced line-probe arrays (LAPs) will be applied to test the susceptibility level of *M. tb* isolates. The inclusion and exclusion criteria are listed below.

#### Inclusion criteria

- Patients diagnosed as active pulmonary TB based on bacteriological results
- Patients willing to receive hospitalized treatment for the first 2 weeks
- Treated with standardized FLDs regimen
- Aged 18–65 years
- Written informed consent

#### Exclusion criteria

- Pregnancy
- Diagnosed gastrointestinal diseases
- Severe liver or renal dysfunction or disease
- History of antibiotic intake in the past 3 months before TB diagnosis
- DST-confirmed MDR (resistant to both INH and RFP)

#### Healthy controls

Healthy controls will be selectively enrolled from the local population during the routine health check-up. The inclusion and exclusion criteria are summarized below.

#### Inclusion criteria

- No active TB disease
- No history of TB
- Aged 18–65 years

#### Exclusion criteria

- Pregnancy
- Diagnosed gastrointestinal diseases
- Severe liver or renal dysfunction or disease
- History of antibiotic intake in the previous 3 months

### Sample size

Because of the absence of prior data on the study aims of the microbiota in a cohort study design of this topic, a pragmatic approach is chosen to determine the sample size. Based on the previous collaboration with the study sites, each site should enroll 50 eligible TB patients every year so that a patient cohort of 300 cases is expected within 2 years. Referred to the treatment failure rate (10%) in China [1], 30 patients in the conducted cohort are estimated to have unfavorable treatment outcomes. The sample size of microbiota study is usually selected empirically, a wide range of study settings indicates that 30 patients each group would likely be sufficient to assess phenotypic heterogeneity at the molecular level [21, 22].

Healthy controls will be equally recruited from the population taking routine health check-up in Sichuan and Shanghai with a total amount of 60. By assuming the rates of intestinal flora dysbiosis are 20% in TB patients [11] and 5% [23] in healthy controls [23], the sample size of 200 TB patients and 60 healthy controls has over 85% power to detect a significant difference in the composition of gut microbiota at the 5% significance level.

### Study procedures

Patients with bacteriologically-confirmed TB are informed about the study by the study coordinators. After receiving the written informed consent, socio-demographic characteristics (date of birth, gender, education, income, etc.), behaviors (alcohol use, smoking, etc.) and clinical information (concomitant medication, physical examination results, TB severity, etc.) of the participants will be documented by a well-designed structured questionnaire.

According to the national guidelines of TB treatment in China, all drug-susceptible TB patients are recommended hospitalization (2 weeks) for the start of TB treatment. The standardized first-line treatment regimen is comprised of 2 months intensive phase and 4–6 months continuation phase. Considering different treatment stages and national regulations, enrolled TB patients will be sampled at Day-0 (important to sample BEFORE treatment), Day-14, the end of intensive-phase (usually at Month-2), Month-5, the end of the treatment and the sixth month after completion of therapy. During treatment, the disease progresses, adverse effects, TB-scores [24], clinical manifestations, drug regimen, and doses of the patients will be documented. Smear microscopy testing is performed at Day-0, Month-2, Month-5 and the end of therapy. DST testing is carried out at inclusion. Additionally, in total six fecal samples will be collected from the patients at inclusion and each scheduled follow-up visit. Subjects in the healthy control arm will have their fecal specimens collected once at inclusion. Questionnaires will be used to record the socio-demographic and behavioral information as well as the health check-up results.

**Fig. 1:**
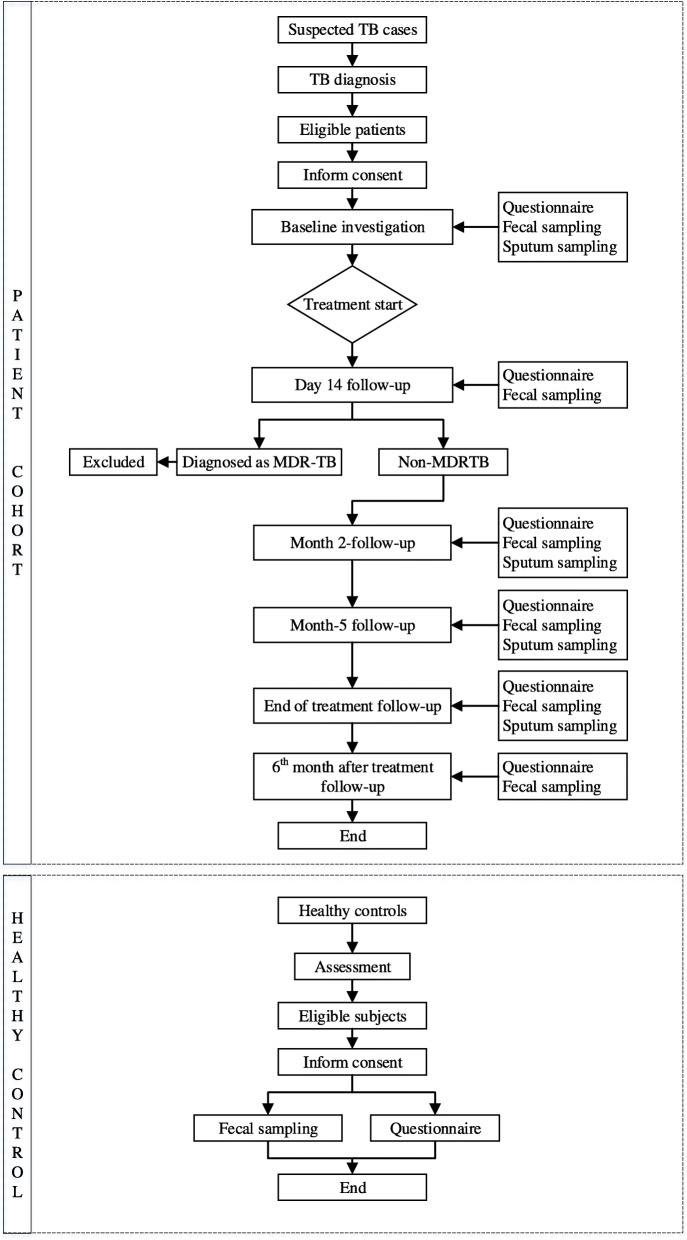
Flow-chart of participants enrollment and follow up. Note: TB, tuberculosis; MDR-TB, multidrug-resistant tuberculosis

The bio-specimen will be transported to the TB laboratory at the designated hospitals or local Centers for Disease Prevention and Control within 12 h after collection. Stool samples should be stored at − 80 °C until DNA extraction. Figure 1 and Table 2 provide an overview of the study flow-chart and the timeline of sample and information collection.

**Table 2:** Summary of data collection and timeline

Timeline	Day-0	Day-14	Month-2	Month-5	End of therapy	6th month after treatment
Demographics						
Age, Sex, Occupation	X					
Financial situation	X					
Health conditions						
Comorbidities	X					
Smoking status	X					
Drinking status	X					
Clinical variables						
TB diagnostic information^a^	X					
Physical examination	X	X	X	X	X	X
Renal and liver function tests	X	X	X	X	X	X
Erythrocyte Sedimentation Rata testing	X	X	X	X	X	X
Sputum smear test	X	X	X	X	X	
TB-Score	X	X	X	X	X	
Chest X-ray	X		X	X	X	
Drug susceptibility test	X					
Current medication	X	X	X	X	X	
Drug dose	X	X	X	X	X	
Adverse effect		X	X	X	X	X
Treatment outcome					X	
Biological specimen collection						
Stool sampling	X	X	X	X	X	X
Sputum sampling	X		X	X	X	

^a^*TB* Tuberculosis

### Laboratory methods

#### Smear microscopy testing

Routine smear microscopy testing is carried out at the laboratory of designated TB hospitals using AFB smear microscopy.

#### Drug susceptibility testing

Routine DST is performed according to the Chinese National Guidelines with the proportion method on Lowenstein-Jensen (LJ) medium (sputum culture-positive), and LAPs (sputum culture-negative) according to WHO’s recommendations.

#### Microbial nucleic acid extraction

Microbial genomic DNA will be extracted from the stool samples using the QIAamp DNA Stool Mini Kit (Qiagen, Hilden, Germany). The DNA extraction should be performed in the Class II biologic safety cabinet. The concentration of genomic DNA in each fecal sample will be quantified using a NanoDrop 2000 spectrophotometer (Thermo Scientific, MA, USA). DNA integrity and sizes are assessed using 1% agarose gel electrophoresis.

#### 16S rRNA gene sequencing

Collected fecal samples will be analyzed by 16S rRNA gene sequencing to obtain the information on microbial composition. A nested polymerase chain reaction (PCR) protocol will be employed to amplify and barcode the 16S hypervariable region V3–V4 (primers: 314F: 5′-CCTACGGGRSGCAGCAG-3′ and 806R: 5′-GGACTACVVGGGTATCTAATC-3′) producing ∼425 base pair PCR product. PCR amplification will be performed in 20 μL reactions containing 10× polymerase mix (Life Technologies, Carlsbad, CA, USA), 10 μM of the forward and reverse primers, and 25 ng of template DNA. The PCR program is set as: initial denaturation at 95 °C for 5 min; 25 cycles of denaturation at 95 °C for 30 s, annealing at 56 °C for 30 s, and extension at 72 °C for 40 s; and final extension of 72 °C for 10 min. The dual-indexed amplicon mixture will be pooled according to the manufacturer’s instructions (Illumina, Inc., San Diego, CA, USA), and then sequenced on the Illumina HiSeq 2500 platform to produce 2 × 250 base pair reads.

### Statistical analysis

#### Descriptive data

Appropriate statistical tests will be applied to analyze and present descriptive data. Comparisons between groups will be performed with Student’s *t*-test and Pearson’s Chi-square test for quantitative and categorical variables, respectively.

#### Microbiome analysis

The raw 16S rRNA gene data will be processed to generate amplicon sequence variants (ASVs) using DADA2 program [25]. Taxonomies will be assigned using Silva [26] as the reference database. Alpha diversity (such as Observed ASVs, Chao1, Shannon, Simpson, etc.) and beta diversity measures will be analyzed using QIIME2 [27] and the Wilcoxon and Kruskal–Wallis tests will be used for statistical testing of two and multi-group comparisons, respectively. Principal coordinate analysis will be used to interrogate the robustness of group-wise clustering, and Adonis test will be used to estimate statistical significance of group-wise beta diversity [27]. Generalized linear model (GLM) will be performed to evaluate the influence factors that may affect the gut microbiota of patients, such as gender, body mass index, TB severity, age, morbidities and so on [28]. Then using GLM to estimate the dynamic changes of microbiota composition with the control of selected confounders [29]. Differential abundance analysis of ASVs will be carried out using STAMP [30] and *p*-values will be adjusted for multiple testing (FDR-adjusted Wald Test). Correlations between clinical parameters and bacterial genera will be calculated using canonical correspondence analysis or redundancy analysis [31]. Random forests approach will be performed to find the most important species as marker taxa correlating with unfavorable treatment outcomes. The data will be analyzed using the QIIME2 pipeline and R [32]. A *p*-value or q-value ≤0.05 will be considered as statistically significant.

### Ethical considerations

The study is an observational study performed in accordance with Good Clinical Practice and the Declaration of Helsinki. Ethical approval (IRB#2018-01-0656) was given by the Institutional Review Board (IRB) at the School of Public Health, Fudan University (FUSPH).. Prior to the study start, study teams of nurses, doctors and laboratory staff participated in training workshops of the study protocol and ethical considerations, led by the main study investigators from FUSPH. All participants sign an informed consent prior to enrolment in the study, or in the case of illiteracy, a fingerprint is given under observation by a witness. For each enrolled subject, a unique study identification number will be used to identify the subject’s information and sample. All the questionnaires and other study documents do not include any identifying information linked to the study code in order to maintain confidentiality for all records and data of the participants.

## Discussion

In this prospective cohort study, we present a comprehensive, multi-dimensional approach to investigate the gut microbiota in TB patients. This might be beneficial and indicative for future studies focusing on the influence of long-term using antibiotics on gut microbiota and its impact on TB treatment. The diverse microbial communities residing in our guts are found to produce bioactive compounds which can modulate host immune responses and influence the occurrence and development of distant organ diseases [3]. However, the effect of anti-TB chemotherapy on microbiome diversity is studied only recently. Reduced bacterial diversity and perturbed microbial community structures have been observed in TB patients with the comparison to healthy individuals [13, 15, 33]. Recently, a longitudinal study [34] was performed in India with an enrollment of six TB patients. The results showed that anti-TB treatment had little overall effect on the diversity of gut microbiota, but the relative abundances of specific taxa were altered after 2 months of treatment. At present, little is known about the association between gut microbiota alterations and clinical outcomes of TB patients. Large-scale and well-designed prospective cohort studies with longer follow-up are urgently needed to help us better understand the long-term use of FLDs on patients’ intestinal microbiota and the impact of gut microbiota alteration on therapeutic efficacy.

The standardized 6–8 months anti-TB treatment course with unified FLDs and prolonged use duration provides a unique opportunity to observe the effects of continuous antibiotics administration on gut microbiota. Selecting objects from different regions, with relatively large sample size and healthy controls, help to increase the representativeness of the study. Sample validity is ensured through a tightly controlled follow-up period and standardized sampling procedures. Another major strength of this study is systematically assessing the influence of administration on the human gut microbiome in a multi-center large cohort of TB patients during their entire treatment. The statistical analysis in this study will be performed from an epidemiological perspective to supplement and validate the study findings. The findings provided by this study will help us better understand the role of gut microbiota and its association with adverse effects and unfavorable treatment outcomes, which could be used to guide and personalize anti-TB treatment in the future.

In conclusion, this study will help us better understand the relationship between gut microbiota and clinical consequences of TB treatment, which will be valuable not only for Chinese TB patients but also applicable to other high TB burden countries. Based on these findings, we will be able to answer whether the gut microbiota has potential implications for the development of targeted therapeutics and better management strategies.

## Data Availability

The datasets used and/or analyzed during the current study are available from the corresponding author on reasonable request.

## Abbreviations

AFB: Acid-fast bacilli
ASVs: Amplicon sequence variants
DST: Conventional drug-susceptibility testing
EMB: Ethambutol
FLDs: First-line anti-TB drugs
FUSPH: School of Public Health, Fudan University.
GDP: Gross domestic product
GLM: Generalized linear model
INH: Isoniazid
IRB: Institutional review board
LAPs: Line-probe arrays
LJ: Lowenstein-Jensen
*M. tb*: *Mycobacterium tuberculosis*
MDR: Multidrug-resistant tuberculosis
PCR: Polymerase chain reaction
PZA: Pyrazinamide
RFP: Rifampicin
rRNA gene: ribosomal ribonucleic acid
WHO: World Health Organization
